# Surfperches versus Damselfishes: Trophic Evolution in Closely Related Pharyngognath Fishes with Highly Divergent Reproductive Strategies

**DOI:** 10.1093/iob/obae018

**Published:** 2024-05-27

**Authors:** W J Cooper, M R Conith, A J Conith

**Affiliations:** Biology Department, College of Science and Engineering, Western Washington University, Bellingham, WA 98225, USA; Marine and Coastal Science, Western Washington University, Bellingham, WA 98225, USA; Biology Department, College of Science and Engineering, Western Washington University, Bellingham, WA 98225, USA; Department of Biology, DePaul University, Chicago, IL 60604, USA

## Abstract

Surfperches and damselfishes are very closely related ovalentarians with large reproductive differences. Damselfishes are typical of most Ovalentaria in that they lay demersal eggs that hatch into small, free-feeding larvae. Surfperches are unusual among ovalentarians and most acanthomorphs in having prolonged internal development. They are born at an advanced stage, some as adults, and bypass the need to actively feed throughout an extended period of ontogeny. Damselfishes and surfperches possess the same modifications of the fifth branchial arch that allow them to perform advanced food processing within the pharynx. This condition (pharyngognathy) has large effects on the evolution of feeding mechanics and trophic ecology. Although the evolution of pharyngognaths has received considerable attention, the effects of different reproductive strategies on their diversification have not been examined. We compared head shape evolution in surfperches and damselfishes using geometric morphometrics, principal component analyses, and multiple phylogenetic-comparative techniques. We found that they have similar mean head shapes, that their primary axes of shape variation are comparable and distinguish benthic-feeding and pelagic-feeding forms in each case, and that, despite large differences in crown divergence times, their head shape disparities are not significantly different. Several lines of evidence suggest that evolution has been more constrained in damselfishes: Head shape is evolving faster in surfperches, more anatomical traits have undergone correlated evolution in damselfishes, there is significant phylogenetic signal in damselfish evolution (but not surfperches), and damselfishes exhibit significant allometry in head shape that is not present in surfperches.

## Introduction

The surfperches (Embiotocidae; Ovalentaria; Acanthomorphata) are a family of viviparous, primarily marine, acanthomorph fishes endemic to the coastal North Pacific (Bernardi and Bucciarelli 1999; Longo and Bernardi 2015). They have internal fertilization and undergo prolonged development within pouches inside their mother's ovaries whence they derive nutrients (Baltz and Knight 1983; Bernardi 2005; Izumiyama et al. 2020). Surfperches are not born until they have reached the equivalent of at least the juvenile stage of most other fishes, and some males are born sexually mature (Baltz and Knight 1983; Schmitt and Holbrook 1984; Schultz 1993; Izumiyama et al. 2020). This mode of reproduction frees them from the need to capture food until very late in development. It is also unusual among acanthomorphs, which account for ∼85% of marine fish diversity (Wainwright and Longo 2017).

**Fig. 1:**
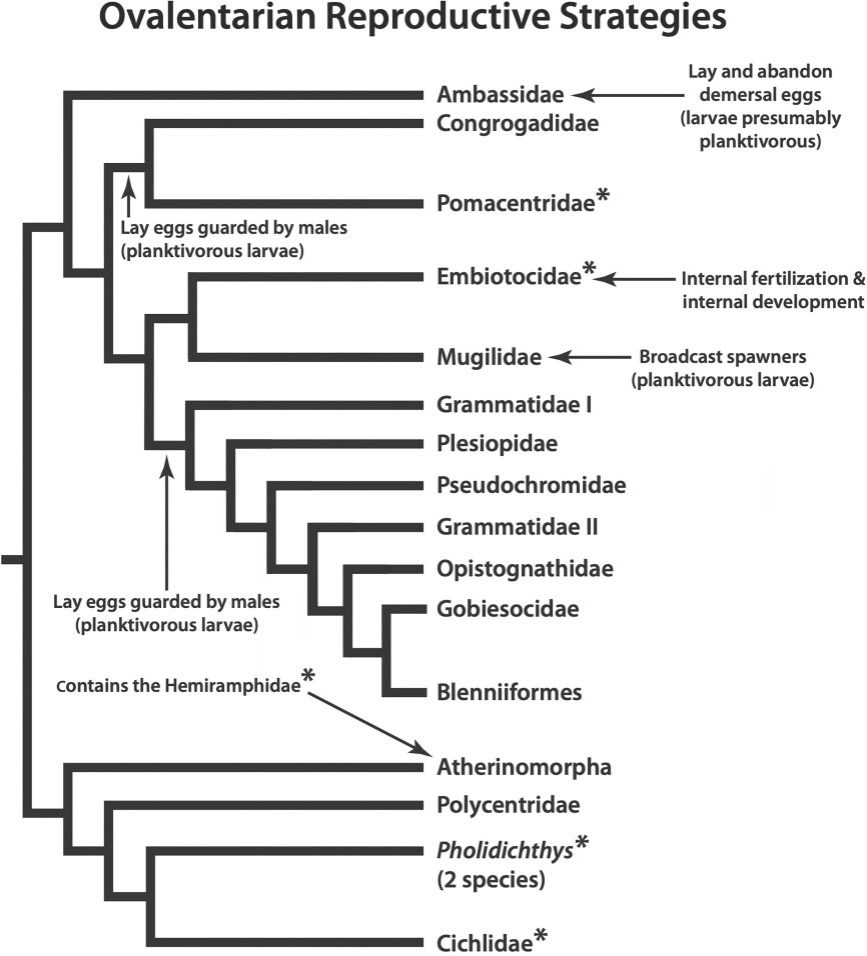
Phylogeny of the Ovalentaria modified from Rabosky et al. (2018), with taxonomy from Betancur-R et al. (2017). Asterisks (*) denote pharyngognath fishes. The primary reproductive modes of the lineages most closely related to surfperches are indicated. Modeled on fig. 1 from Thieme et al. (2022).

Like surfperches, the closely related damselfishes (Pomacentridae; Fig. 1) are primarily marine, nearshore fishes, but they have a markedly different developmental strategy. Damselfishes are similar to most other acanthomorphs in that they begin feeding at an early stage, typically as small planktonic larvae that prey upon smaller zooplankton (Sampey et al. 2007; Carassou et al. 2009; Jackson and Lenz 2016). Unlike their larger conspecifics, small fish larvae experience high water viscosities as they move and feed (i.e., they live in a low Reynolds number environment; Hernandez 1995, 2000; China et al. 2017; Galindo et al. 2019; Sommerfeld and Holzman 2019). They tend to use very fast and extensive head and jaw motions that are consistent with the need to overcome high resistance when drawing food into the mouth via suction (Hernandez 1995, 2000; Holzman et al. 2015; Galindo et al. 2019; Cooper et al. 2020).

The functional needs of early developmental stages can constrain the evolutionary diversification of adult morphologies, functional abilities, and ecology (Jm 1984; Zelditch et al. 1993; Klingenberg 2005; Cooper and Steppan 2010; Cooper et al. 2020). Here, we explore the hypothesis that surfperches have been released from developmental constraints on their adult trophic evolution that are imposed when small larvae must actively feed. Internal fertilization is unusual among the Ovalentaria (Fig. 1), and, indeed, the name of this taxon is based on the fact that laying demersal eggs that adhere to the substrate is ancestral for the lineage (Wainwright et al. 2012), and the production of small, zooplanktivorous larvae is pervasive among all species closely related to surfperches (Fig. 1; Near et al. 2012, 2013; Wainwright et al. 2012; Rabosky et al. 2018). The damselfishes provide an opportunity to compare diversification in surfperches with evolution among fishes that not only have strong differences in development but also represent the only closely related lineage to share a fundamental aspect of their trophic anatomy that has been shown to strongly affect trophic diversification: pharyngognathy (Fig. 1; McGee et al. 2015, 2016; Conith and Albertson 2021; Roberts-Hugghis et al. 2023).

Pharyngognath fishes possess modifications of the fifth branchial arch that allow efficient processing of engulfed food items. The fifth ceratobranchials on either side of the ventral pharynx have been fused into a single element (the lower pharyngeal jaw) that can be adducted against the upper pharyngeal jaws (primarily composed of the second and third infrapharyngobranchials; left and right sides unfused) via a muscle sling (the fourth levator externus; Liem 1973). The upper pharyngeal jaws also articulate with the cranium via derived basipharyngeal joints (Liem 1973). Although many fishes have specialized fifth branchial arches that contribute to feeding, these additional modifications have allowed pharyngognaths to become especially proficient at food processing (McGee et al. 2015). Multiple studies have shown that pharyngognathy heavily influences diversification in both feeding ecology and oral jaw morphology (McGee et al. 2015, 2016; Conith and Albertson 2021; Roberts-Hugghis et al. 2023). Surfperches and damselfishes represent the two most closely related pharyngognath lineages that have diversified into a number of genera and species, and have substantial differences in their reproductive biology (Fig. 1).

**Fig. 2:**
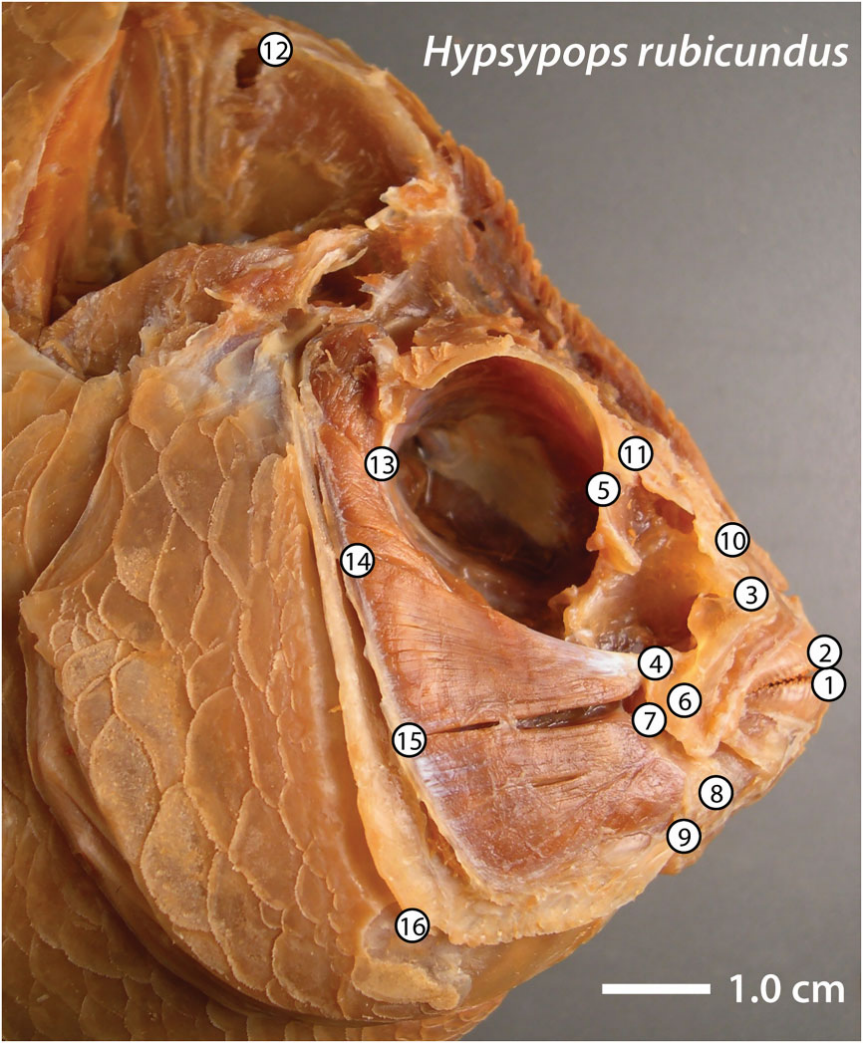
Anatomical landmarks used in shape analyses (anatomy after Barel et al. 1976; Datovo and Vari 2013): (1) tip of the anterior-most tooth on the dentary; (2) tip of the anterior-most tooth on the premaxilla; (3) maxillary–palatine joint; (4) insertion of the *pars malaris* division of the *adductor mandibulae* on the maxilla; (5) ventral tip of the preorbital process; (6) maxillary–articular joint (also posterior tip of the dentigerous process of the premaxilla); (7) insertion of the *pars rictalis* division of the *adductor mandibulae* on the primordial process of the articular; (8) articular–quadrate joint; (9) insertion of the interopercular ligament on the articular; (10) posterior tip of the ascending process of the premaxilla; (11) joint between the nasal bone and the neurocranium; (12) dorsalmost tip of the supraoccipital crest on the neurocranium; (13) most posteroventral point of the eye socket; (14) most dorsal point on the origin of the *pars malaris* division of the *adductor mandibulae* on the preopercular; (15) most dorsal point on the origin of the *pars rictalis* division of the *adductor mandibulae* on the preopercular; and (16) posteroventral corner of the preopercular.

We sought to determine whether the functional morphology of feeding has evolved at different rates and/or in a different manner in surfperches and damselfishes. To do this, we examined important aspects of their trophic anatomy (Fig. 2) within a phylogenetic context. We compared their extant head shape disparity and rates of head shape evolution. Slower evolutionary rates are more consistent with the presence of constraints than fast rates. We also tested for the presence of phylogenetic signal (also an indication of constraint) in their adult head shape variation. Significant evidence of allometry (i.e., covariation between body size and shape) can indicate that developmental processes are constraining shape diversification (Voje et al. 2014). We therefore tested for the presence of allometric signal among the morphological data from both groups.

Extensive morphological and functional diversification along a bentho-pelagic ecological axis has been described for damselfishes and cichlids (Cichlidae), which are another ovalentarian pharyngognath lineage (Cooper and Westneat 2009; Cooper et al. 2010, 2017; Conith and Albertson 2021). Because these previous studies examined head shape data similar to those collected here, the current work provided an opportunity to examine broad patterns of pharyngognath diversification relevant to over 2000 species. We therefore compared the major axes of morphological variation in surfperches and damselfishes to determine whether they have diversified along similar ecomorphological gradients. The primary axes of head shape in both cichlids and damselfishes strongly distinguish benthic-feeding fishes from those that feed primarily from the water column (pelagic feeding). We therefore tested for differences in the functional morphology of feeding between benthic-feeding and pelagic-feeding species.

## Methods

### Specimens

Specimens of surfperches and damselfishes were obtained from the fish collections of The Burke Museum (Seattle, WA, USA), The California Academy of Sciences (San Francisco, CA), The Field Museum (Chicago, IL, USA), The Scripps Institution of Oceanography (La Jolla, CA, USA), and the laboratory collection of W.J. Cooper (Western Washington University, Bellingham, WA, USA). We examined three specimens each from 14 of the 23 described embiotocid species (Eschmeyer and Fricke 2023), with representatives from 12 of the13 genera (the monotypic genus *Hypocritichthys* was not examined) and wide coverage across the phylogeny (Longo and Bernardi 2015; Longo et al. 2018). We examined 44 pomacentrid species, with representatives from 27 of the 29 genera (*Altrichthys* and *Plectroglyphidodon* were not examined) and wide coverage across the phylogeny (McCord et al. 2021; Tang et al. 2021; Eschmeyer and Fricke 2023). All pomacentrid genera sampled were represented by at least three specimens, except for the monotypic *Nexilosus* (one specimen). Three specimens each were examined for >70% of the pomacentrid species included in the study. See Supplementary Table S1 for a list of all species examined.

We dissected heads to expose anatomical landmarks (LMs) of functional importance to fish feeding (Fig. 2). Our references to cranial bone anatomy follow Barel et al. (1976). For muscular anatomy, we follow Datovo and Vari (2013).

### Collection of shape data

We used geometric morphometrics to quantify anatomical shape. We obtained the coordinate locations of 16 anatomical LMs of functional importance to feeding from digital images of dissected heads (Fig. 2). The StereoMorph package (Olsen and Westneat 2015), which runs in the R programming environment (R Core Team 2020), was then used to quantify anatomical shape by determining the coordinate locations of LMs.

We used the geomorph package in R (Baken et al. 2021) to perform a generalized Procrustes analysis to remove the effects of size, translation, and rotation from each set of landmark coordinates. For species represented by multiple specimens, we then calculated a Procrustes mean head shape configuration for each.

### Tree structure used in evolutionary analyses

For all phylogenetic-comparative analyses, we used a pruned maximum likelihood tree from Rabosky et al. (2018) that included all species in our dataset.

### Analyses of shape

To visualize head shape variation among surfperches and damselfishes as captured by the LM data described above, we performed principal component analyses (PCAs) and visualized relationships between taxa in shape space by projecting the phylogeny onto the shape data, as in a phylomorphospace analyses, which are PCAs with the phylogenies projected onto the shape data (Rohlf 2002; Sidlauskas 2008). The first principal component calculated using these methods represents the largest source of variation in a dataset regardless of phylogenetic relationships between specimens. In order to compare overall morphological variation between damselfishes and surfperches, we determined the degree of alignment between the first component of the damselfish and surfperch PCAs, as detailed by Friedman et al. (2022). Briefly, we used R to calculate the standardized pairwise angle between the first components of the damselfish and surfperch PCAs. Angle values could vary between 0 and 90, with smaller values indicating increasingly similar axes of variation. We then determined significance by simulating traits under a model of Brownian motion (*n* = 1000) using the function mvSIM in the mvMORPH package in R (Clavel et al. 2015).

### Tests for correlated evolution among aspects of trophic anatomy

To compare patterns of evolution in the functional morphology of feeding between surfperches and damselfishes, we determined the amount of evolutionary covariation between 11 anatomical traits important to feeding in both lineages (Table 1; see below) using a phylogenetic generalized least-squares (PGLS) regression. This was achieved using the gls function in the nlme package in R (Pinheiro et al. 2022).

**Table 1:** PGLS results for surfperches and damselfishes

**Surfperches**											
Head area	NA										
Jaws area	0.264	NA							0.566		
Eye area*	0.432	0.117	NA		0.283		0.206	0.417	−0.122	−0.037	
Biting muscle area	0.805	0.173	0.856	NA							
Supraoccipital crest area	0.061	0.931	**0.042†**	0.802	NA						
Lower jaw biting in-lever	0.055	0.497	0.967	0.158	0.207	NA					
Lower jaw biting out-lever*	0.666	0.252	**0.006**	0.710	0.685	0.218	NA	0.455	0.624	0.776	0.220
Lower jaw biting MA	0.517	0.545	**0.016†**	0.421	0.203	0.073	**0.021†**	NA		−0.044	
Premaxilla ascending arm length	0.148	**0.010**	**0.049**	0.818	0.850	0.234	**0.002†**	0.113	NA	0.487	
Premaxilla dentigerous arm length*	0.190	0.091	**0.048**	0.620	0.897	0.314	**<0.001†**	**0.032**	**0.001†**	NA	0.081
Premaxilla ascending arm length/dentigerous arm length	0.581	0.812	0.359	0.182	0.907	0.871	**0.031**	0.114	0.591	**0.003**	NA
**Damselfishes**											
Head area*	NA		0.646			0.654	0.154	0.580	0.192	0.381	
Jaws area	0.297	NA						0.566			0.554
Eye area	**0.001**	0.070	NA	0.601	0.283				0.471		
Biting muscle area	0.172	0.240	**0.007**	NA							
Supraoccipital crest area	0.412	0.068	**0.010†**	0.530	NA						
Lower jaw biting in-lever*	**<0.001**	0.591	0.189	0.687	0.802	NA	0.533	0.446	0.195	0.734	
Lower jaw biting out-lever*	**0.003**	0.301	0.653	0.276	0.552	**<0.001**	NA	0.455	0.624	0.776	
Lower jaw biting MA	**<0.001**	**0.020**	**0.021†**	0.501	0.544	**0.002**	**0.033†**	NA			
Premaxilla ascending arm length*	**0.001**	0.089	0.704	0.317	0.544	**0.002**	**<0.001†**	0.767	NA	0.487	0.817
Premaxilla dentigerous arm length*	**<0.001**	0.933	0.751	0.376	0.962	**<0.001**	**<0.001†**	0.291	**<0.001†**	NA	
Premaxilla ascending arm length/dentigerous arm length	0.771	**0.019**	0.939	0.459	0.544	0.682	0.433	0.780	**<0.001**	0.474	NA
	Head area	Jaws area	Eye area	Biting muscle area	Supraoccipital crest area	Lower jaw biting in-lever	Lower jaw biting out-lever	Lower jaw biting MA	Premaxilla ascending arm length	Premaxilla dentigerous arm length	Premaxilla ascending arm length/dentigerous arm length

*Note. P*-values below the diagonal, *r*-values above. Significant *P*-values shaded/bold. *r*-values shown for significant correlation only. Correlation between pairs of traits that were significant for both lineages (†). Groups of traits that have undergone correlated evolution with each other within a lineage (*). Head area adjusted by standard length (SL). Jaws, eye, biting muscle, and supraoccipital crest areas adjusted by head area.

We used the ImageJ software program (Schneider et al. 2012) to calculate the areas and lengths of the 11 anatomical traits analyzed with PGLS from images of dissected heads taken in profile with the mouths closed (Table 1; Fig. 2). We calculated area measurements from outlines of the following structures: the entire head (see below), jaws (the combined area of the mandible, maxilla, and premaxilla), eye (outer edge of the eye socket), superficial biting muscles (the *pars malaris* and *pars rictalis* divisions of the *adductor mandibulae*), and the supraoccipital crest. The outline of the head was traced from a point on the dorsal surface of the head immediately above LM 12, along the posterior edge of the supraoccipital crest to the posterodorsal corner of the subopercle, then along the posterior edge of the operculum (the posterior edges of the opercle and subopercle) to a point on the ventral surface immediately below the posteroventral corner of the subopercle, then continuing anteriorly to LM 1, then to LM 2, and then posteriorly along the dorsal surface until connecting with the starting point above LM 12. The length of the lower jaw biting in-lever was measured between the dorsalmost point of insertion of the *pars rictalis* on the primordial process of the articular in the lower jaw (LM 7) and the articular–quadrate joint (i.e., the lower jaw joint; LM 8). The length of the lower jaw biting out-lever was measured between LMs 1 and 8. The mechanical advantage (MA) applied by the *pars rictalis* to the lower jaw during biting (lower jaw biting MA) was measured as the ratio of the lower jaw biting in-lever to the lower jaw biting out-lever. The anatomical characteristics of lower jaw in- and out-levers for fish biting and the associated MA are adapted from Westneat (1994). The length of the ascending arm of the premaxilla (distance between LMs 2 and 10) is a major determinant of maximum upper jaw protrusion distance in acanthomorph fishes (Motta 1984; Osse 1985; Cooper et al. 2017). The dentigerous (tooth-bearing) arm of the premaxilla lies adjacent to the dorsal surface of the lower jaw when the mouth is closed, and its length was measured from LM 2 to the posterior tip of this process (typically close to LM 8). The relative lengths of the ascending and dentigerous arms of the premaxilla are associated with the ratio of jaw protrusion distance to maximum gape distance, which can be an important determinant of a fish's ability to produce suction (Gibb and Ferry-Graham 2005).

### Comparisons of head shape disparity

We assessed differences in head shape disparity between clades using the morphol.disparity function in the R package geomorph (Baken et al. 2021). This function estimates differences in disparity by first calculating the Procrustes variance using residuals obtained from a PGLS estimation of coefficients and measures the distance of each individual in a clade from the mean shape for that clade (Zelditch et al. 2012). Head shapes were calculated from the positions of the 16 LMs depicted in Fig. 2. Permutation procedures are used to test for significance between clades, and a null distribution is generated by randomizing the vectors of shape residuals 1000 times among clades (Adams and Collyer 2015).

### Rates of morphological evolution

We compared rates of head shape evolution between surfperches and damselfishes using the compare.evol.rates function in the R package geomorph to assess differences in the rates of head shape evolution. This method uses a species distance approach to calculate a phylogenetically corrected rate of morphological evolution (σ^2^) under Brownian motion for both clades (Adams 2014c). The ratio of rates between clades is used as a test statistic, and differences between clades are determined by comparing the observed shape data to a distribution of tip data simulated 1000 times under a Brownian motion model of evolution that uses a common rate for all species.

### Phylogenetic signal

We estimated phylogenetic signal in both head shape and overall body size in surfperches and damselfishes using the physignal function in the geomorph package in R. This function permutes data under a model of Brownian motion (1000 iterations) and calculates a multivariate Blomberg's *K* statistic that is used to determine whether the data are significantly different from a null model in which there is no difference between clades (Blomberg et al. 2003; Adams 2014a).

Given the differences in the sample sizes of our two families (damselfish, *n* = 44; surfperch, *n* = 14), estimation of evolutionary parameters in the surfperches could result in high error rates depending on the structure of the data (Boettiger et al. 2012). To determine whether we had the statistical power to detect differences in our surfperch data, we performed a phylogenetic power analysis using the phylocurve package in R (Goolsby 2016).

### Allometry

To assess the effect of allometry in our cranial shape data, we performed a phylogenetically corrected multivariate regression of shape on centroid size using the procD.pgls function in geomorph (Adams 2014b). We assessed allometry in each clade independently to examine whether there was significant covariation between size and shape (i.e., evidence of significant allometry) in either clade (Adams 2014b). Evidence of significant allometry is a potential indicator that aspects of development have constrained the anatomical evolution of a lineage (Voje et al. 2014).

### Tests for differences in head shape between clades and among ecotypes

We used phylogenetic analysis of variance (pANOVA) to examine anatomical LM data (Fig. 2) to determine whether species head shapes differed by clade or aspects of feeding ecology (Table 2). These analyses were performed using the procD.pgls function from the geomorph package, and *post hoc* comparisons were assessed using the pairwise function from the RRPP package in R (Baken et al. 2021). First, we evaluated whether head shapes differ between damselfishes and surfperches. Next, we grouped species by feeding locations (i.e., benthic, pelagic, or benthic/pelagic; benthic/pelagic = substantial pelagic and benthic feeding; see Supplementary Table S1) and tested for head shape differences between ecotypes both within clades and across a combined dataset of surfperches and damselfishes. For the feeding locations of damselfishes, we follow McCord et al. (2021). The feeding locations of surfperches were determined from the literature (see citations in Supplementary Table S1).

**Table 2:** Surfperch diet categories and results of pANOVA tests for differences in head shape

Species	Diet 1	Diet 2	Feeding mode
*Amphistichus argenteus*	Benthic invertebrates	Echinoderms and crabs	Durophagy
*Brachyistius frenatus*	Zooplankton and benthic invertebrates	**Zooplankton**	**Suction**
*Cymatogaster aggregata*	Zooplankton and benthic invertebrates	Amphipods	Picking
*Ditrema temminckii*	Amphipods	Amphipods	Picking
*Embiotoca caryi*	Benthic invertebrates	Amphipods	Winnowing
*Embiotoca jacksoni*	Amphipods	Amphipods	Winnowing
*Hyperprosopon argenteum*	Amphipods	Amphipods	Picking
*Hysterocarpus traskii*	Zooplankton and benthic invertebrates	Amphipods	Picking
*Micrometrus aurora*	Algae, insects, and amphipods	Algae, insects, and amphipods	Biting and picking
*Neoditrema ransonnetii*	**Zooplankton**	**Zooplankton**	**Suction**
*Phanerodon furcatus*	Benthic invertebrates	Amphipods	Durophagy
*Phanerodon vacca*	Molluscs	Amphipods	Durophagy
*Rhacochilus toxotes*	Benthic invertebrates	Amphipods	Winnowing
*Zalembius rosaceus*	Benthic invertebrates	Amphipods	Picking
	Procrustes ANOVA results (top row): *F*-values above *P*-values; significant *P*-values*		
	The results of significant post hoc, pairwise comparison are in the bottom three rows		
	2.211	2.321	1.46
	0.008*	0.010*	0.037*
	**Zooplankton** vs. amphipods *P* = 0.002*	**Zooplankton** vs. amphipods *P* = 0.003*	**Suction** vs. durophagy *P* = 0.042*
	**Zooplankton** vs. benthic invertebrates *P* = 0.012*	**Zooplankton** vs. echinoderms and crabs *P* = 0.008*	**Suction** vs. picking *P* = 0.042*
	**Zooplankton** vs. zooplankton and benthic invertebrates *P* = 0.042*		**Suction** vs. winnowing *P* = 0.001*

*Note*. In each column, the species within the categories marked in bold had significantly different head shapes from those within the categories marked by shaded cells. Significant differences denoted by an asterisk (*).

The results of tests for differences in trophic anatomy between damselfishes with different diets have been published previously (Cooper et al. 2017). Here, we performed similar tests for surfperches. Because fish diets do not always fall within discrete categories, we assigned surfperch species to two diet classes for pANOVA to perform a more rigorous examination. We also grouped surfperches by foraging method based on the literature (see citations in Supplementary Table S1). These groupings include the winnowing technique used by many surfperches and other fishes to sift food from mouthfuls of substrate (Drucker and Jensen 1991; Wainwright et al. 2004; Hernandez and Staab 2015).

## Results

### Patterns of head shape variation in surfperches and damselfishes

**Fig. 3:**
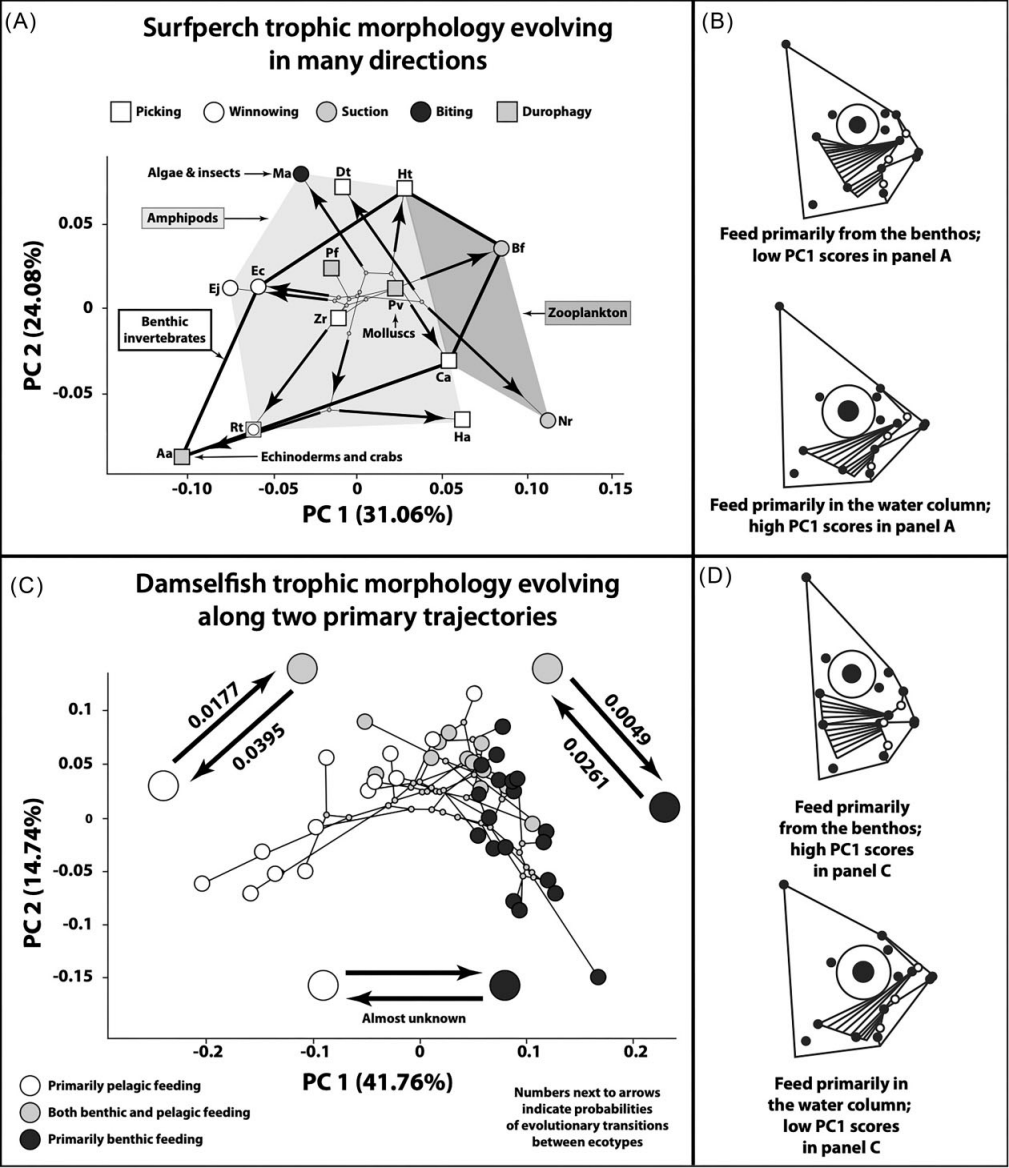
Patterns of head shape variation in surfperches and damselfishes. (**A**) Phylomorphospace of surfperch head shape. Polygons link and enclose species that consume substantial amounts of a specific food type. Areas of overlap indicate utilization of multiple food types. Additional diet information is provided for three species: *M. aurora, Amphistichus argenteus* (Agassiz 1854), and *Phanerodon vacca* (Girard 1854). Mechanisms of acquiring food are denoted by species symbols (key near top of panel). *Rhacochilus toxotes* (Agassiz 1854) employs both winnowing and durophagy. Species key: Aa (*A. argenteus*); Bf (*Brachyistius frenatus*); Ca (*Cymatogaster aggregata*); Dt (*Ditrema temminckii*); Ec (*Embiotoca caryi*); Ej (*E. jacksoni*); Ha (*Hyperprosopon argenteum*); Ht (*Hysterocarpus traskii*); Ma (*M. aurora*); Nr (*Neoditrema ransonnetii*); Pf (*Phanerodon furcatus*); Pv (*P. vacca*); Rt (*R. toxotes*); and Zr (*Zalembius rosaceus*). (**B**) Visual characterization of the head shape variation described by PC1 in panel **A**. As PC1 scores decrease or increase, species head shapes become more similar to the upper or lower image, respectively. Circles denote the 16 anatomical LM locations analyzed. Open circles denote joints between bones. Two divisions of the *adductor mandibulae* are denoted by the two groups of converging lines located below the eye. (**C**) Phylomorphospace of damselfish head shape (see Cooper et al. 2017 for detailed species distributions). Probabilities for evolutionary transitions between damselfish ecotypes from McCord et al. (2021). (**D**) Visual characterization of the head shape variation described by PC1 in panel **C**.

**Fig. 4:**
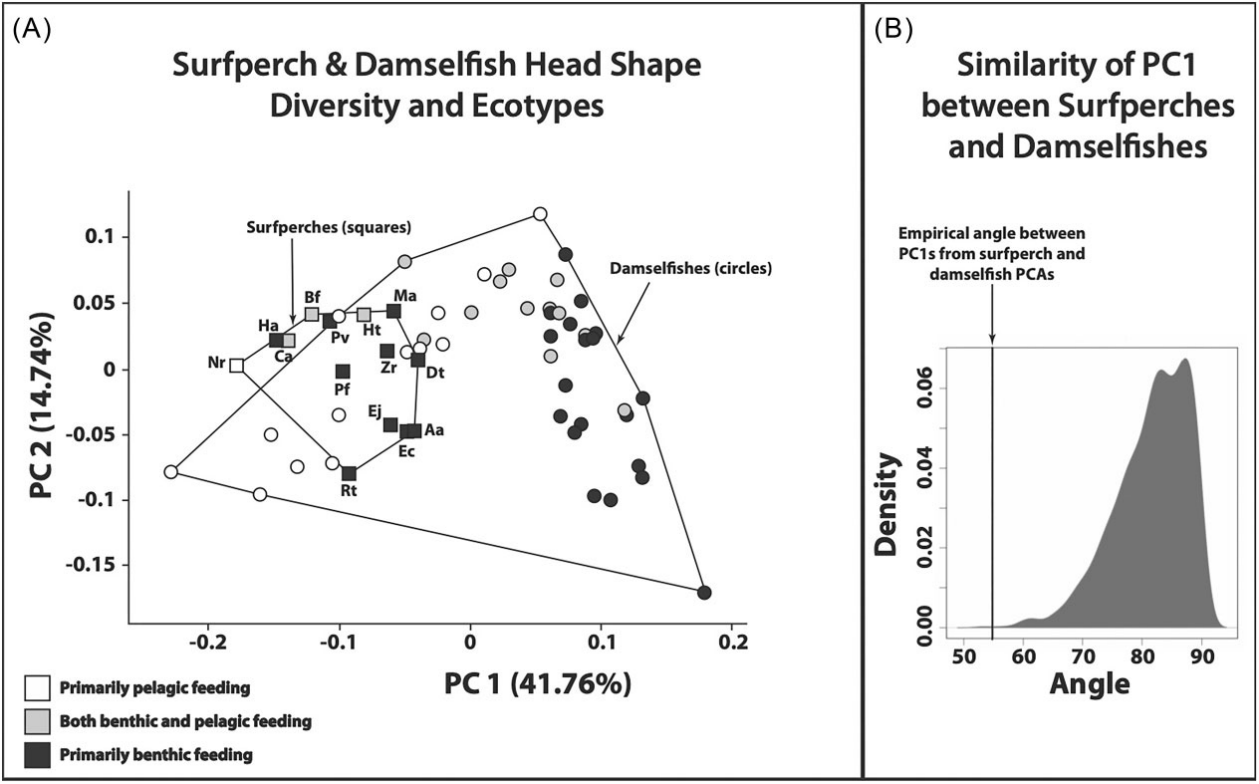
Comparisons of trophic evolution in surfperches and damselfishes. (**A**) Principal component score plot derived from a PCA of both surfperch and damselfish head shape data. Polygons enclose all members of a lineage. (**B**) Results of a simulation test (1000 iterations) using a Brownian motion model that demonstrates that the angle between PC1 calculated from the surfperch data and PC1 calculated from the damselfish data is significantly smaller than would be expected by chance (i.e., the orientation of PC1 is highly similar for these datasets).

The results of PCAs indicate that surfperches and damselfishes exhibit similar patterns of head shape variation, especially along PC1, which is largely characterized by differences in jaw length, the length of the ascending arm of the premaxilla, head depth, the size of the supraoccipital crest, biting muscle anatomy (size, length, and orientation), eye size, and dorsoventral eye placement for both families (Fig. 3). Indeed, the angle between the first principal components derived from separate PCAs of the surfperches and damselfishes is smaller than expected under a model of Brownian motion (empirical θ = 54.9°; distribution of simulated θ = 53.1°–90.0°; *P* = 0.001; Fig. 4B). This suggests that their primary axes of morphological variation, while not completely aligned as would be expected with θ = 0°, have been expanding along similar evolutionary trajectories.

When surfperch and damselfish head shape data were analyzed using separate PCAs, benthic-feeding and pelagic-feeding fishes were found to occupy different ends of their respective PC1 axes. This pattern was also present in the PCA score plot derived from a combined analysis of shape data from both families (Fig. 4A). Although benthic-feeding and pelagic-feeding fishes from the same lineage had higher and lower PC1 scores relative to each other, all surfperches were found to have head shapes more like pelagic-feeding than benthic-feeding damselfishes (Fig. 4A).

### More extensive correlated evolution among trophic characters in damselfishes

The results of PGLS analyses found significant evolutionary correlation among more anatomical traits in damselfishes than in surfperches (Table 1). For damselfishes, the PGLS results identified 20 cases of significant correlations that involve all 11 of the traits examined. Conversely, in surfperches, we found 13 significant correlations among only eight of these traits (Table 1).

Five traits have undergone correlated evolution with each other during damselfish evolution, and four traits have done so during surfperch evolution (Table 1). Within these two intercorrelated groups of traits, three were common to both lineages: the length of the lower jaw out-lever that is applied during biting (lower jaw biting out-lever), and the lengths of the ascending and dentigerous arms of the premaxilla (Table 1). Head area has undergone correlated evolution with six other traits in damselfishes, but with no other traits in surfperches (Table 1). In surfperches, we see evidence for the coordinated evolution of eye area with multiple aspects of jaw anatomy and bite mechanics, while in damselfishes, eye area has undergone coordinated evolution with only one aspect of bite mechanics: the MA employed by the lower jaw during biting (lower jaw biting MA; Table 1). Also in surfperches, the length of the lower jaw in-lever that is applied during biting (lower jaw biting in-lever) has not undergone significantly correlated evolution with any of the other traits, whereas it has undergone correlated evolution with five other traits in damselfishes (Table 1). Surprisingly, biting muscle area has undergone correlated evolution with only one other trait in the damselfishes and with no other traits in surfperches (Table 1).

### Similar degrees of head shape diversity, but different rates of anatomical evolution

Although damselfishes exhibit greater variation in head shape relative to surfperches (Fig. 4A), the differences in shape disparity were not significant (*P* = 0.123; surfperch head shape disparity = 0.013, damselfish head shape disparity = 0.020). Likewise, the pANOVA results showed no significant difference between damselfish and surfperch mean head shapes (*P* = 0.987). Head shape is evolving significantly faster in surfperches than in damselfishes (*P* = 0.05; surfperch σ^2^ = 2.32, damselfish σ^2^ = 1.73).

### Lower levels of constraint on surfperch head shape evolution

There is evidence of significant phylogenetic signal in damselfish head shape evolution (*K*_mult_ = 0.75, *P* = 0.001) but not in surfperch evolution (*K*_mult_ = 0.63, *P* = 0.289). We found no evidence of significant phylogenetic signal for body size in either group (damselfishes: *K*_mult_ = 1.90, *P* = 0.935; surfperches: *K*_mult_ = 4.28, *P* = 0.371). A power analysis revealed that analysis is likely robust to low sample size in the surfperch, as we found the analysis exhibited high power to detect differences (null log-likelihood [LL] = −7178, alternative LL = −5457; LL ratio = 3442; critical test statistic = 414.7; power = 1, *P* < 0.001).

### Allometric signal in the evolution of damselfishes, but not surfperches

We found significant evidence of allometry (significant covariation between head shape and body size) in damselfishes (*P* = 0.001). Differences in body size account for 14.5% of the morphological variation in damselfish head shapes. We did not find significant covariation between head shape and body size in surfperches (*P* = 0.307).

### Benthic feeders and water column feeders have different head shapes in both lineages

The results of quantitative analyses of head shape were consistent with the qualitative PCA findings. pANOVA results show that feeding location (benthos, water column, or both) is a significant predictor of head shape in both lineages regardless of the dataset analyzed: surfperches only (*P* = 0.036); damselfishes only (*P* = 0.001); and combined dataset (*P* = 0.001). Pairwise comparisons revealed that head shape is distinct between benthic and pelagic feeders in all datasets: surfperches (*P* = 0.001); damselfishes (*P* = 0.001); and combined (*P* = 0.001). Head shapes were distinct between benthic and intermediate feeders (those that feed from both locations) in the damselfish (*P* = 0.011) and combined datasets (*P* = 0.022) but not in the surfperch dataset (*P* = 0.813). The head shapes of pelagic and intermediate feeders were not significantly different for any of the datasets, though values approached significance for the damselfish and combined datasets (surfperches *P* = 0.812; damselfishes *P* = 0.062; combined *P* = 0.059).

In regard to surfperch diets and feeding strategies, significant differences in head shape were only seen between surfperch species that primarily use suction to capture zooplankton from the water column and those that acquire most of their prey from the benthos (e.g., amphipods, molluscs, echinoderms, crabs; Table 2) by winnowing, picking (e.g., plucking small invertebrates from the substrate), or durophagy (consuming hard-shelled prey). *Micrometrus aurora* (Jordan and Gilbert 1880), which is unusual among surfperches in consuming benthic algae and insects as well as amphipods (Hubbs 1921; Boyle and Horn 2006), always occupied its own diet or feeding mode category (Table 2). It was the only species whose head shape was never found to be significantly different from those of other surfperches (Table 2).

## Discussion

The head shapes of surfperches have evolved much more freely than those of damselfishes. This evolution has been significantly faster, and their cranial variation is not significantly different from that of pomacentrids despite a wide gap in the initial divergence times of the crown lineages (13–18 mya for surfperches; 55.5 mya for damselfishes; Longo and Bernardi 2015; McCord et al. 2021). Damselfishes also exhibit strong evidence of phylogenetic signal in their head shape variation, while surfperches do not. Furthermore, the existence of significant covariation between body size and head shape (i.e., significant allometry) in damselfishes suggests that their cranial diversification may be constrained by developmental processes that impose specific patterns of anatomical integration (Voje et al. 2014). Under such circumstances, increased shape variation tends to evolve in conjunction with an expansion in body size, but the appearance of new morphologies that do not retain ancestral size/shape relationships arises less frequently (Voje et al. 2014).

Surfperch head shapes are radiating in many directions (Fig. 3A), while those of damselfishes are primarily transitioning back and forth along two trajectories (Fig. 3C). Recent work by McCord et al. (2021) has shown that pomacentrids, which feed jointly from the benthos and the water column (benthic/pelagic-feeding), frequently give rise to both pelagic-feeding and benthic-feeding forms, with transitions rates of 0.0395 and 0.0261, respectively, but that shifts from pelagic-feeding or benthic-feeding back to benthic/pelagic-feeding are much less common (transitions rates of 0.0177 and 0.0049, respectively; Fig. 3C). Transitions between benthic-feeding and pelagic feeding ecotypes are almost unknown (McCord et al. 2021).

**Fig. 5:**
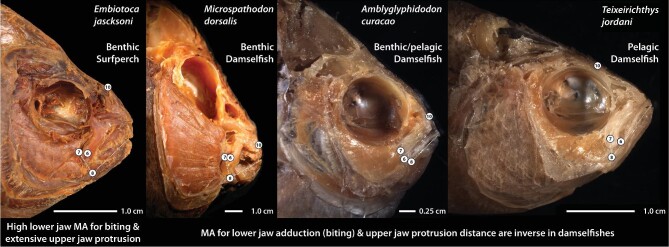
Head morphologies of a benthic-feeding surfperch capable of both high bite forces (large biting muscles and high lower jaw MA) and extensive jaw protrusion, and representatives of the three damselfish ecotypes in which bite force and jaw protrusion ability are inverse to each other. Aspects of morphology that have undergone highly integrated evolution in both damselfishes and surfperches are depicted: length of the dentigerous process of the premaxilla (distance from LM 6 to tip of the upper jaw); length of the ascending process of the premaxilla (distance from LM 10 to tip of the upper jaw); length of the in-lever for adducting the lower jaw (distance from LM 7 to LM 8); length of the out-lever for the lower jaw (distance from LM 8 to tip of the lower jaw); eye area; and head area. Lower jaw MA = in-lever length/out-lever length. For landmark identity, see Fig. 2.

There are higher levels of evolutionary integration among aspects of damselfish cranial anatomy in comparison to surfperches. Twenty anatomical traits have evolved together in damselfishes as opposed to 13 in surfperches (Table 1). Four traits have evolved with each other in surfperches, and five have done so in damselfishes (Table 1; Fig. 5). In both lineages, the lengths of the ascending and dentigerous arms of the premaxilla have evolved with the length of the lower jaw out-lever, and in damselfishes these three traits have also evolved with the length of the lower jaw in-lever. The ratio of the ascending and dentigerous processes of the premaxilla is an important determinant of jaw protrusion ability, and the ratio of the lower jaw's in-lever and out-lever determines the MA applied to it during biting (Fig. 5).

Pelagic-feeding damselfishes use extensive jaw protrusion to rapidly expand the buccal cavity and generate suction for capturing elusive zooplankton such as copepods (Cooper et al. 2017). Jaw protrusion is much more limited in benthic-feeding damselfishes that rely more upon biting than suction during feeding (Cooper et al. 2017). Larger lower jaw MAs confer more efficient force transmission and promote benthic feeding using hard bites. Smaller lower jaw MAs confer more efficient motion transfer and promote pelagic feeding using fast bites. Bite force and protrusion ability have evolved together and in an inverse manner as damselfishes transitioned back and forth between benthic/pelagic feeding and pelagic feeding, and between benthic/pelagic feeding and benthic feeding. This pattern of integration can promote transitions between a limited number of niches along a bentho-pelagic ecological axis, but the path that damselfishes follow as they do this appears to be tightly constrained (Figs. 3C and 5).

The past decade has seen a profound shift in how integration is thought to affect evolvability. Although there was not complete consensus, integration was previously expected to largely constrain evolution (Bjorklund 1996; Schluter 1996; Klingenberg 2005; Goswami et al. 2014). Both modeling and empirical studies of multiple lineages now indicate that high levels of integration can promote adaptive evolution along specific phenotypic trajectories (Hu et al. 2016; Felice et al. 2018; Du et al. 2019; Evans et al. 2021; Burns et al. 2023; Knapp et al. 2023). Evidence from phylogenetic-comparative work with several highly successful fish radiations has provided particularly strong support for this hypothesis (Hu et al. 2016; Du et al. 2019; Evans et al. 2021; Burns et al. 2023; Knapp et al. 2023). The emerging view is that integration will promote evolvability when phenotypic trajectories align with adaptive lines of least resistance (Felice et al. 2018). This can occur when patterns of trait covariation imposed by developmental processes constrain phenotypes to those that promote fitness across a range of ecological niches (Marroig et al. 2009; Goswami et al. 2014; Felice et al. 2018; Du et al. 2019; Burns et al. 2023). This perspective translates Schluter's (1996) concept of adaptation along genetic lines of least resistance into a phenotypic context.

The lengths of the ascending and dentigerous arms of surfperch premaxilla have evolved in correlation with the length of the lower jaw's out-lever, but not the length of its in-lever (Table 1). Since it is the ratio of in-lever to out-lever length that determines the lower jaw bite MA (Westneat 1994), these aspects of surfperch premaxilla shape are not evolving with this determinant of bite force. Surfperches should therefore be able to evolve their bite forces and jaw protrusion abilities with a higher degree of independence in comparison to damselfishes (Fig. 5). This is likely to have contributed to their ability to evolve head shapes both along a greater number of trajectories (Fig. 3A) and more rapidly than damselfishes. It is notable that multiple benthic-feeding surfperches that either acquire prey by winnowing or remove algae from substrates also exhibit substantial jaw protrusion (e.g., *Embiotoca jacksoni* and *M. aurora*; Drucker and Jensen 1991; Boyle and Horn 2006), a combination that is unknown in damselfishes (Fig. 5).

That both surfperches and damselfishes are pharyngognaths is an important aspect of the comparisons made here (Fig. 1). Karel Liem's classic prediction that the pharyngeal jaws of cichlid fishes (Cichlidae) contributed significantly to their evolutionary diversification (Liem 1973) has stimulated multiple investigations of pharyngognathy as a key innovation over the past half century. The development of extensive, well-supported molecular phylogenies for acanthomorph fishes and advances in phylogenetic-comparative analytical methods greatly facilitated this work. Evolutionary examinations of pharyngognathy in cichlids, surfperches, damselfishes, hemiramphids, and the more distantly related wrasses have now demonstrated that, although the functional abilities of their pharyngeal jaws have had a significant effect on their evolution, this effect was not exactly as Liem predicted. Although pharyngognathy promotes diversification into feeding niches where extensive food processing is required (e.g., certain types of durophagy and herbivory), it also seems to act as a constraint (McGee et al. 2015, 2016; Roberts-Hugghis et al. 2023). Pharyngognaths are much less likely to become piscivores than nonpharyngognaths, and rather than acting as largely independent components of the overall feeding mechanism, there appears to be a high level of integration between pharyngeal jaws and oral jaws at both the morphological and genetic levels (McGee et al. 2015, 2016; Conith and Albertson 2021; Roberts-Hugghis et al. 2023). This integration may act to constrain rather than promote oral jaw evolution so that, contrary to Liem's prediction, the result of pharyngognathy can be a reduction in the rate of oral jaw diversification.

There is a consistent pattern to the evolution of trophic morphology among multiple ovalentarian phyaryngognath lineages (surfperches, damselfishes, and cichlids; Fig. 1). In all cases, the largest axis of head shape variation strongly distinguishes benthic-feeding forms that use high bite forces and shorter jaws from pelagic-feeding forms that use faster bite speeds and longer jaws (Figs. 3 and 4; Cooper and Westneat 2009; Cooper et al. 2010, 2017). The diversification of East African rift-lake cichlids has repeatedly proceeded along this axis during each of their massive radiations in Lakes Tanganyika, Malawi, and Victoria (Cooper et al. 2010; Corn et al. 2021). This same pattern has also been described previously for damselfishes, and here we see that not only do surfperches exhibit a similar distribution in shape space (Figs. 3A and 4A) but the primary axes of head shape variation are also parallel for both embiotocids and pomacentrids (Fig. 4A and B). These patterns are likely reinforced by the basic differences in the bite forces and bite speeds required for benthic feeding and pelagic feeding, and the fact that there is typically a trade-off between force and speed and between mouth size and MA in the feeding mechanics employed by acanthomorphs (Arnold et al. 2011; Corn et al. 2021), though this trade-off is not inherently universal in such systems (McHenry and Summers 2011). The supposition that the bite mechanics of damselfishes and surfperches are subject to such trade-offs is reinforced by the finding that there are significant differences in the functional morphology of feeding between pelagic-feeding and benthic-feeding species in both clades (Table 2; Cooper et al. 2017).

## Conclusion

Whether or not their evolutionary differences are driven by divergent reproductive strategies, the patterns of head shape diversification described by surfperches and damselfishes exhibit strong differences. The younger surfperch clade has radiated faster and in many directions. Damselfish evolution has been more constrained and shows a higher level of integration, and the probabilities of transition between their limited numbers of ecotypes are unequal. Benthic/pelagic-feeding damselfishes invade specialized niches (either pelagic- or benthic-feeding) more frequently than specialists evolve into species with generalized diets. Unlike damselfishes, surfperches do not exhibit an inverse relationship between jaw protrusion and utilization of benthic food items and this may have facilitated their trophic evolution. These two lineages demonstrate that adaptive diversification in trophic form, function, and ecology can describe very different patterns in closely related pharyngognaths.

## Supplementary Material

obae018_Supplemental_File
