# Supplementary material for: Surfperches versus Damselfishes: Trophic Evolution in Closely Related Pharyngognath Fishes with Highly Divergent Reproductive Strategies
Source: Integr Org Biol. 2024 May 27;6(1):obae018. doi: 10.1093/iob/obae018 (PMC11210498; doi:10.1093/iob/obae018)
Supplement: obae018_Supplemental_File [file obae018_supplemental_file.docx]

Supplementary Table 1. Species examined, number of specimens each, their primary feeding locations (B=Benthic; P=Pelagic, water column; B/P=Benthic & Pelagic), size, and feeding references.

| **Species** | **Family** | **Number of specimens** | **Primary feeding location** | **Maximum body size (SL; cm)** | **Sources for feeding data** |
| --- | --- | --- | --- | --- | --- |
| *Amphistichus argenteus* | E | 3 | B | 43.0 | Barry et al. 1996 (and references therein) |
| *Brachyistius frenatus* | E | 3 | B/P | 22.0 | Bray and Ebeling 1975 |
| *Cymatogaster aggregata* | E | 3 | B/P | 20.3 | Woods 2010 |
| *Ditrema temminckii* | E | 3 | B | 24.0 | Hayase and Tanaka 1980 |
| *Embiotoca caryi* | E | 3 | B | 30.0 | Ebling and Laur 1986 |
| *Embiotoca jacksoni* | E | 3 | B | 39.0 | Schmitt and Holbrook 1984; Barry et al. 1996; Ebling and Laur 1986 |
| *Hyperprosopon argenteum* | E | 3 | B | 30.0 | Hobson and Chess. 1976. |
| *Hysterocarpus traskii* | E | 3 | B/P | 15.0 | Turner 1966; Baltz and Moyle 1981 |
| *Micrometrus aurora* | E | 3 | B | 18.0 | Boyle and Horn 2006; Hubbs 1921 |
| *Neoditrema ransonnetii* | E | 3 | P | 13.0 | Hayase and Tanaka 1980 |
| *Phanerodon furcatus* | E | 3 | B | 32.0 | Bray and Ebeling 1975: Barry et al. 1996 |
| *Phanerodon vacca* | E | 3 | B | 44.2 | Brett 1979; Boulding et al. 2001;  Ebling and Laur 1986 |
| *Rhacochilus toxotes* | E | 3 | B | 47.0 | Ebling and Laur 1986 |
| *Zalembius rosaceus* | E | 3 | B | 20.0 | Allen 1982 |
| *Abudefduf bengalensis* | P | 1 | B | 17.0 | McCord et al. 2021 (and references therein) |
| *Abudefduf saxatilis* | P | 3 | B | 22.9 | McCord et al. 2021 (and references therein) |
| *Abudefduf septemfasciatus* | P | 1 | B | 23.0 | McCord et al. 2021 (and references therein) |
| *Abudefduf sexfasciatus* | P | 1 | B/P | 19.0 | McCord et al. 2021 (and references therein) |
| *Abudefduf sordidus* | P | 1 | B | 24.0 | McCord et al. 2021 (and references therein) |
| *Abudefduf taurus* | P | 1 | B | 25.0 | McCord et al. 2021 (and references therein) |
| *Abudefduf whitleyi* | P | 1 | B/P | 14.0 | McCord et al. 2021 (and references therein) |
| *Acanthochromis polyacanthus* | P | 3 | B/P | 14.0 | McCord et al. 2021 (and references therein) |
| *Amblyglyphidodon curacao* | P | 3 | B/P | 11.0 | McCord et al. 2021 (and references therein) |
| *Amblyglyphidodon leucogaster* | P | 1 | P | 13.0 | McCord et al. 2021 (and references therein) |
|  |  |  |  |  |  |
| **Species** | **Family** | **Number of specimens** | **Primary feeding location** | **Maximum body size (SL; cm)** | **Sources for feeding data** |
| *Amblypomacentrus breviceps* | P | 3 | P | 8.5 | McCord et al. 2021 (and references therein) |
| *Amblypomacentrus tricinctus* | P | 1 | B/P | 6.0 | McCord et al. 2021 (and references therein) |
| *Amphiprion akindynos* | P | 3 | B/P | 9.0 | McCord et al. 2021 (and references therein) |
| *Amphiprion biaculeatus* | P | 3 | P | 17.0 | McCord et al. 2021 (and references therein) |
| *Azurina hirundo* | P | 3 | P | 14.0 | McCord et al. 2021 (and references therein) |
| *Azurina multilineata* | P | 1 | P | 20.0 | McCord et al. 2021 (and references therein) |
| *Cheiloprion labiatus* | P | 3 | B | 6.0 | McCord et al. 2021 (and references therein) |
| *Chromis punctipinnis* | P | 3 | P | 25.0 | McCord et al. 2021 (and references therein) |
| *Chromis weberi* | P | 3 | P | 13.5 | McCord et al. 2021 (and references therein) |
| *Chrysiptera brownriggii* | P | 1 | P | 8.0 | McCord et al. 2021 (and references therein) |
| *Chrysiptera cyanea* | P | 3 | B/P | 8.5 | McCord et al. 2021 (and references therein) |
| *Chrysiptera oxycephala* | P | 3 | P | 9.0 | McCord et al. 2021 (and references therein) |
| *Dascyllus melanurus* | P | 3 | B/P | 8.0 | McCord et al. 2021 (and references therein) |
| *Dischistodus melanotus* | P | 3 | B | 16.0 | McCord et al. 2021 (and references therein) |
| *Hemiglyphidodon plagiometopon* | P | 3 | B | 18.0 | McCord et al. 2021 (and references therein) |
| *Hypsypops rubicundus* | P | 3 | B | 30.0 | McCord et al. 2021 (and references therein) |
| *Lepidozygus tapeinosoma* | P | 3 | P | 10.5 | McCord et al. 2021 (and references therein) |
| *Mecaenichthys immaculatus* | P | 3 | B | 12.0 | McCord et al. 2021 (and references therein) |
| *Microspathodon dorsalis* | P | 3 | B | 31.0 | McCord et al. 2021 (and references therein) |
| *Neoglyphidodon melas* | P | 2 | B | 18.0 | McCord et al. 2021 (and references therein) |
| *Neoglyphidodon nigroris* | P | 3 | B | 13.0 | McCord et al. 2021 (and references therein) |
| *Neoglyphidodon oxyodon* | P | 3 | B | 15.0 | McCord et al. 2021 (and references therein) |
| *Neoglyphidodon thoracotaeniatus* | P | 1 | B/P | 13.5 | McCord et al. 2021 (and references therein) |
| *Neopomacentrus taeniurus* | P | 3 | P | 10.0 | McCord et al. 2021 (and references therein) |
| *Nexilosus latifrons* | P | 1 | B | 30.0 | McCord et al. 2021 (and references therein) |
| *Parma microlepis* | P | 3 | B | 14.0 | McCord et al. 2021 (and references therein) |
|  |  |  |  |  |  |
| **Species** | **Family** | **Number of specimens** | **Primary feeding location** | **Maximum body size (SL; cm)** | **Sources for feeding data** |
| *Pomacentrus alexanderae* | P | 3 | B/P | 9.0 | McCord et al. 2021 (and references therein) |
| *Pomachromis richardsoni* | P | 3 | P | 6.0 | McCord et al. 2021 (and references therein) |
| *Pristotis obtusirostris* | P | 3 | P | 14.0 | McCord et al. 2021 (and references therein) |
| *Pycnochromis amboinensis* | P | 3 | P | 10.0 | McCord et al. 2021 (and references therein) |
| *Similiparma hermani* | P | 3 | B/P | 16.0 | McCord et al. 2021 (and references therein) |
| *Stegastes flavilatus* | P | 3 | B | 10.0 | McCord et al. 2021 (and references therein) |
| *Stegastes lacrymatus* | P | 3 | B | 10.0 | McCord et al. 2021 (and references therein) |
| *Teixeirichthys jordani* | P | 3 | P | 14.0 | McCord et al. 2021 (and references therein) |

**References**

Allen, M. J. 1982. Functional structure of soft-bottom fish communities of the Southern California Shelf. Ph.D., University of California, San Diego.

Baltz, D. M. & Moyle, P. B. 1981. Morphometric Analysis of Tule Perch (*Hysterocarpus traski*) Populations in 3 Isolated Drainages. Copeia, 305-311.

Barry, J. P., Yoklavich, M. M., Cailliet, G. M., Ambrose, D. A. & Antrim, B. S. 1996. Trophic ecology of the dominant fishes in Elkhorn Slough, California, 1974-1980. Estuaries, 19, 115-138.

Boulding, E. G., Pakes, D. & Kamel, S. 2001. Predation by the pile perch, *Rhacochilus vacca*, on aggregations of the gastropod *Littorina sitkana*. Journal of Shellfish Research, 20, 403-409.

Boyle, K. S. & Horn, M. H. 2006. Comparison of feeding guild structure and ecomorphology of intertidal fish assemblages from central California and central Chile. Marine Ecology Progress Series, 319, 65-84.

Bray, R. N. & Ebeling, A. W. 1975. Food, activity, and habitat of three "picker·type" microcarnivorous fishes i n the kelp forests off Santa Barbara, California. United States Fisheries Bulletin, 73, 815-829.

Brett, J. R. 1979. Some Morphological and Behavioral Adaptations of Pile Perch (*Rhacochilus vacca*) Feeding on Mussels (*Mytilus edulis*). Canadian Journal of Zoology-Revue Canadienne De Zoologie, 57, 658-664.

Ebeling, A. W. & Laur, D. R. 1986. Foraging in Surfperches - Resource Partitioning or Individualistic Responses. Environmental Biology of Fishes, 16, 123-133.

Hayase, S. & Tanaka, S. 1980. Feeding Ecology of 3 Species of Embiotocid Fishes in the *Zostera marina* Belt of Odawa Bay. Bulletin of the Japanese Society of Scientific Fisheries, 46, 1469-1476.

Hobson, E. S. & Chess , J. R. 1976. Trophic interactions among fishes and zooplankters near shore at Santa Catalina Island, California. Fishery Bulletin, 74, 567-598.

Hubbs, C. L. 1921. The Ecology and Life-History of *Amphigonopterus aurora* and of Other Viviparous Perches of California. Biological Bulletin, 40, 181-209.

Mccord, C., Nash, C., Cooper, W. & Westneat, M. 2021. Phylogeny of the damselfishes (Pomacentridae) and patterns of asymmetrical diversification in body size and feeding ecology. PLoS ONE, 16, e0258889.

Schmitt, R. J. & Holbrook, S. J. 1984. Ontogeny of Prey Selection by Black Surfperch *Embiotoca jacksoni (*Pisces, Embiotocidae) - the Roles of Fish Morphology, Foraging Behavior, and Patch Selection. Marine Ecology-Progress Series, 18, 225-239.

Turner, J. L. 1966. Distribution of threadfin shad, *Dorosoma petenense*; tule perch, *Hysterocarpus traskii*; sculpin spp. and crayfish spp. in the Sacramento-San Joaquin Delta. California Fisheries Bulletin, 136, 160-167.

Woods, P. J. 2010. Geographic variation in lower pharyngeal jaw morphology in the Shiner Perch *Cymatogaster aggregata* (Embiotocidae, Teleostei). Environmental Biology of Fishes, 88, 153-168.
